# Modular Virus-like Particles for Antigen Presentation: Comparing Genetic Fusion and Click-Chemistry for Purification

**DOI:** 10.3390/ijms262010036

**Published:** 2025-10-15

**Authors:** Karsten Balbierer, Volker Jenzelewski, Fabian C. Herrmann, Michael Piontek, Joachim Jose

**Affiliations:** 1 ARTES Biotechnology GmbH, 40764 Langenfeld, Germany; karsten.balbierer@uni-muenster.de (K.B.); volker.jenzelewski@artes-biotechnology.com (V.J.); michael.piontek@artes-biotechnology.com (M.P.); 2 Institute of Pharmaceutical and Medicinal Chemistry, University of Münster, 48149 Münster, Germany; 3 Institute of Pharmaceutical Biology and Phytochemistry, University of Münster, 48149 Münster, Germany; shermanic@uni-muenster.de

**Keywords:** virus-like particles (VLPs), hepatitis B core antigen (HBc), click-chemistry, antigen presentation

## Abstract

The recent SARS-CoV-2 pandemic has highlighted the need for quickly adaptable technologies in vaccine manufacturing. This can be achieved through virus-like particles (VLPs) as presentation platforms for target antigens. In this study, we investigated the purification of VLPs of the Hepatitis B Core antigen (HBc) and the SplitCore (SplCo) technology. The outer surface protein C (OspC) of *Borrelia burgdorferi* was genetically fused to HBc and its N-terminal SplCo protein. Product solubility in *E. coli* increased from 40% for HBc-OspC to 90% for SplCo-OspC. This could not be reproduced with similar SARS-CoV-2 receptor-binding domain fusions due to inclusion body formation. Hydrophobicity was found to be significantly lowered for the OspC fusions, in particular for the SplCo variant. Pre-purified samples were generated by precipitating soluble cell lysate. Subsequently, solubilized precipitates were subjected to anion exchange chromatography (AEX), and the elution fractions obtained contained VLPs, albeit with low purity. The VLPs were also disassembled prior to AEX for dissociative purification, but a subsequent reassembly could not be achieved for both fusion variants. A novel HBc variant was constructed for post-translational modification via click-chemistry. The solubility and hydrophobicity of this HBc variant remained high, but native AEX resulted in complete product loss. By contrast, a yield of 84% VLPs was obtained for the modified HBc after dissociative AEX. The surface-exposed azide group on the particles, introduced for click-chemistry, enabled coupling to fluorophores without compromising VLP stability. Conjugation efficiencies of up to 59% were obtained. These results suggest the potential of combining HBc and click-chemistry for future applications, e.g., the presentation of immunogenic epitopes or antigens. This underlines that for every antigen, both the optimal scaffold-decoration strategy and the subsequent manufacturing process should be carefully selected.

## 1. Introduction

Virus-like particles (VLPs) have grown in importance in medical and biotechnological applications. VLPs are multiprotein complexes that mimic natural viruses. Clinical approval and licensing of medical applications of VLPs started nearly half a century ago [1]. Advantages of VLPs include their non-infectivity, high stability, optional multivalency, and effective immune stimulation, as well as simple recombinant production and biological safety [2]. At present, they are applied to vaccines, therapeutics, and diagnostic imaging, among others [3,4]. Approved VLP-based vaccines are for Hepatitis B virus (HBV) infections (e.g., Engerix and Heplisav B) and Human Papillomavirus (HPV) infections (e.g., Gardasil and Cervarix) [5]. The Mosquirix Malaria vaccine [6] marks a milestone in the application of chimeric VLPs. It consists of a fusion protein of the self-assembling Hepatitis B surface antigen and a portion of a *Plasmodium falciparum* sporozoite surface protein. Vaccine candidates based on the Hepatitis B core antigen (HBc), which forms the capsid protein of HBV, have undergone several clinical trials. NASVAC has recently passed a clinical phase IIa study as a vaccine for treating chronic Hepatitis B [7]. Further chimeric HBc VLP vaccine candidates are under review, targeting widespread diseases like influenza and foot-and-mouth disease [8,9].

Cost effectiveness, scalability, and robustness of up- and downstream processes are central factors for market competitiveness and clinical approval of new vaccines [10,11]. However, the recent SARS-CoV-2 pandemic has emphasized that modern manufacturing requires quickly adaptable platform technologies and modular vaccine design [12]. In this respect, chromatographic methods for the purification of HBc and other VLPs have gained importance. In this context, anion exchange chromatography (AEX) is a faster and easier-to-scale alternative to methods like preparative size exclusion chromatography (SEC) and centrifugal separations, like sucrose cushion ultracentrifugation (SUC) [13]. HBc self-assembles into spherical structures with a diameter of approximately 30 nm. Disassembly into dimeric subunits during purification enables the removal of impurities like encapsulated host nucleic acids, and subsequent reassembly has been found to improve particle homogeneity [14]. Efforts to understand the assembly mechanism of HBc have revealed nucleation-like kinetics. Trimerization of dimers is followed by the addition of subunits until a complete capsid is formed [15,16]. The main interaction site for these inter-dimer interactions has been identified as a hydrophobic pocket [17]. This pocket can be utilized for hydrophobic interaction chromatography (HIC) to recover particles [18], which may eliminate the need for a separate reassembly step. Purifications using native AEX and AEX under dissociating conditions with subsequent HIC are compared in this study.

Antigen presentation on the surface of HBc VLPs can be achieved primarily through genetic protein fusions in vivo or chemical conjugation in vitro. Fusion proteins possess a covalent peptide bond between each HBc monomer and the antigen. The insertion site in the exposed immunodominant loop, also known as the c/e1-domain or major immunodominant region (MIR), is well studied, and modifications of this area should not directly affect assembly competence [19]. However, inserting larger peptides or proteins can cause folding incompatibilities. Previous research has identified several HBc fusions that cannot be expressed in a soluble form [20]. The SplitCore technology was developed to improve solubility and reduce steric incompatibilities. In short, the HBc monomer is split into two peptide chains between the Pro79 and Ala80 of the MIR, with both subunits co-expressed in equimolar amounts under the control of the same promoter. The antigen is attached to one of the two subunits at its N- or C-terminus, and particle assembly has been shown to still occur [21,22]. However, incompatibilities with particular antigens indicate the need for alternative conjugation methods [23]. Affinity-tag-based approaches have been successfully applied, like the SpyTag/SpyCatcher-System [24] or the SUMO-tag, as applied in the VelcroVax-System [25]. In the present study, a potentially more universal reaction mechanism is proposed using click-chemistry [26]. Copper-catalyzed azido-alkyne cycloaddition (CuAAC) was applied due to its high reactivity and controllable reaction conditions [27]. A free azide and a free alkyne form a covalently bound 1,2,3-triazole ring in the presence of Cu(I)-Ions, which serve as catalysts. Clickable groups can be integrated into proteins by non-natural amino acids (nnAAs), such as azidohomoalanine (AHA) [28]. The CuAAC of VLPs was tested through AHA integration into VLPs, conjugating them to fluorescent dyes with alkyne moieties, but these HBc VLPs were found to decompose during the reaction [29]. The methionines at positions 1 and 66 of HBc were replaced by AHA. These are located in the assembly domain, critical for inter-dimer reactions, which may have been disrupted by the formation of the 1,2,3-triazole ring. For more site-specific nnAA integration, modified tRNA/tRNA-aminoacyl-synthetase pairs have been developed to translate stop-codons in *Escherichia coli* (*E. coli*) to nnAAs [30]. To integrate *p*-azidophenylalanine (*p*AzF), a defined expression vector has been constructed [31]. In this way, *p*AzF can be selectively introduced into the polypeptide chain using the amber stop codon in the encoding region of the expression plasmid. This enables directed integration into the MIR of HBc, leading to surface exposure of the azide-bearing side-chain. Strain-promoted azide–alkyne cycloaddition (SPAAC) has been reported as a copper-free alternative to CuAAC, with comparably high reaction rates [32,33]. Following a similar mechanism, the high ring tension of a cyclooctyne ring facilitates the spontaneous formation of a 1,2,3-triazole ring in the presence of a nearby azide. Reports of successful fluorescent labeling of VLP scaffolds using SPAAC have been published [34]; however, to the best of our knowledge, functionalization of HBc VLPs with this reaction has not been attempted.

Here, we investigate limitations of the SplitCore technology in scalable particle purification based on two representative antigens as fusion partners. The first is the outer surface protein C (OspC) of *Borrelia burgdorferi*, which causes Lyme Disease, transmitted by ticks. Its fusion to HBc, leading to fully assembled chimeric VLPs, has been described previously [19,22]. Additionally, the receptor binding domain (RBD) of the SARS-CoV-2 Spike protein was chosen to validate the suggested advantages of SplitCore. We present an alternative approach for HBc modification using the click-handle *p*AzF (HBc-*p*AzF). This enables chemical conjugation of the antigen by targeting the azide group, potentially optimizing its presentation. A purification pathway combining the advantages of HBc disassembly and reassembly and chromatographic methods such as AEX and HIC has been developed to generate pure and homogeneous HBc VLPs. CuAAC and SPAAC are used to validate the functional accessibility of *p*AzF and investigate the effects of triazole ring formation on the structural stability of VLPs. Finally, variations in the reactivity of SPAAC throughout the purification process are quantified to evaluate the influence of potential contaminants and conformational states.

## 2. Results

### 2.1. Construction of Protein Variants

**Figure 1:**
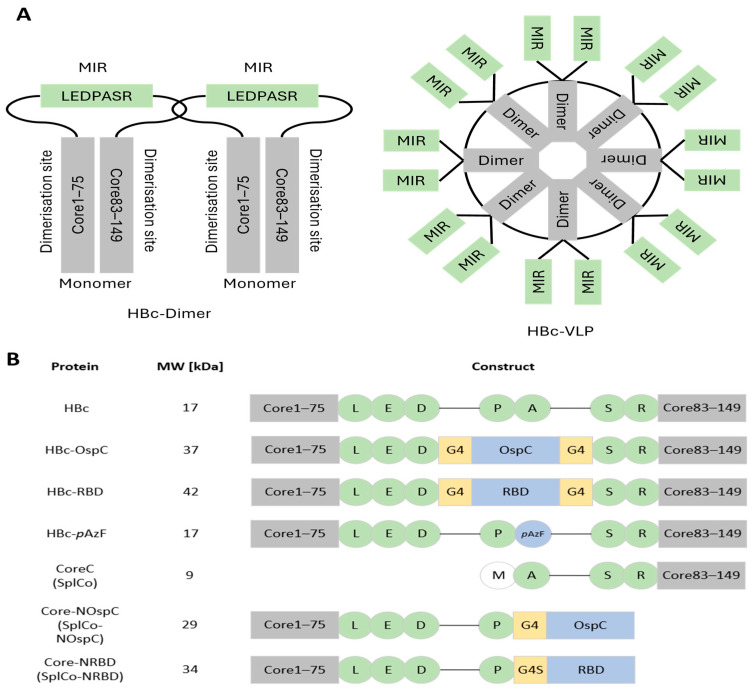
Schematic overview of the functional organization of the protein constructs. (**A**) Organization of the HBc monomers and simplified visualization of the dimer-orientation in a VLP. Protein spikes consisting of two MIRs protrude from the spherical surface of the VLP, while the helical dimerization sites of the HBc protein, crucial for particle assembly, are located proximal to the capsid shell. (**B**) Overview of the different protein variants, their molecular weight (MW), and their position in the insertion site. Protein regions are visualized as blocks, amino acids as circles. Gray and green visualization is used if native to the HBc protein, yellow for linker regions and blue for foreign insertions.

The MIR of the HBc protein was used as the insertion site for all variants in this study. The region with the protein sequence LEDPASR, also known as the assembly domain, connects both dimerization sites of the monomer in a flexible protein loop. After assembling the HBc-dimers into VLPs, the MIRs of each monomer appear on the particle surface as protruding spikes (Figure 1A). For the contiguous chain fusion variants HBc-OspC and HBc-RBD, the foreign antigen sequences were inserted in place of Pro79 Ala80, along with linker regions, to increase structural flexibility. In their SplitCore counterparts, the antigen sequences were C-terminally attached to Pro79, forming the N-terminal half of the SplitCore system (SplCo-NOspC, SplCo-NRBD). They were co-expressed with the CoreC protein, which started at Ala80 of the MIR, after a methionine for translation initiation. For the alternative approach using click-chemistry instead of direct antigen fusions, the nnAA *p*AzF was expressed as an exchange to Ala80 in the variant HBc-*p*AzF (Figure 1B).

### 2.2. Expression and Solubility

#### 2.2.1. Inducible Expression of HBc-*p*AzF and Genetic Fusion Variants

**Figure 2:**
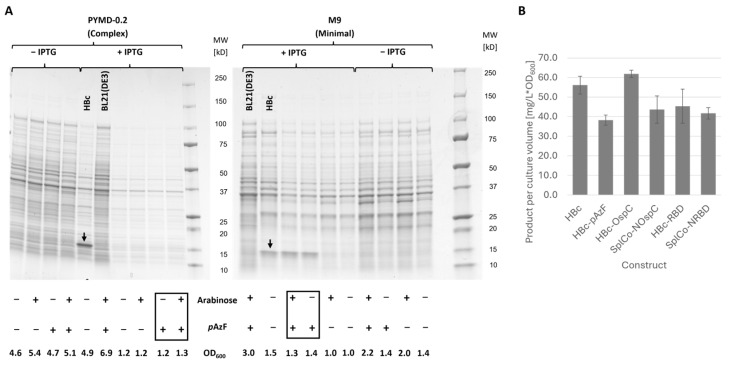
(**A**) SDS-PAA gels (Coomassie, gray-scale) of media comparison and investigation of *p*AzF-Integration into HBc-*p*AzF. Cell harvests of the same volume were chemically lysed in SDS-PAGE sample buffer and directly applied. Black arrows indicate product band of HBc as positive control at 17 kDa. Untransformed BL21(DE3) was cultivated in identical conditions as negative control. Variations in expression media as well as addition of IPTG, *p*AzF and arabinose were investigated, and the OD_600_ of harvested culture suspensions are stated as a measure of saturation growth density. Black boxes highlight the combination of additives for which complete expression of HBc-*p*AzF was expected. (**B**) Comparison of mean product yields for the different protein constructs. Cultures of the HBc-RBD and SplCo-NRBD variants were harvested 4 h post induction to avoid product degradation, all other constructs 17 h post induction. *p*AzF was added after induction for HBc-*p*AzF only. Underlying values were determined by densitometric evaluation of respective product bands after SDS-PAGE of cell harvests in triplicates and normalized to OD_600_ of harvested cell suspensions. A BSA dilution series was used as a calibration standard. For SplCo-variants, only the N-terminal product band was quantified and the CoreC protein was assumed to be present in equimolar amounts and total product calculated accordingly. Error bars represent standard deviation from mean value.

In PYMD-0.2 medium, no product band was detectable after adding IPTG and *p*AzF. A 17 kDa protein band corresponding to the product was only observed in M9 medium, and only when *p*AzF was added during induction (Figure 2A). Culture growth was not impacted by *p*AzF. In the absence of IPTG, arabinose increased the final cell density. However, it did not affect expression levels in the induced cultures. HBc, HBc-OspC, and SplCo-NOspC were expressed under the same conditions, but without the addition of *p*AzF. HBc-RBD and SplCo-NRBD were harvested four hours after induction, as prolonged expression resulted in product degradation or even the complete loss of detectable product. Expression levels varied between 38.2 mg/L and 61.9 mg/L after cell density normalization (Figure 2B), with HBc-*p*AzF showing the lowest and HBc-OspC the highest. The remaining investigated fusion variants showed no notable differences in expression levels in this evaluation.

#### 2.2.2. Recovery of Soluble Product After Mechanical Cell Lysis

**Figure 3:**
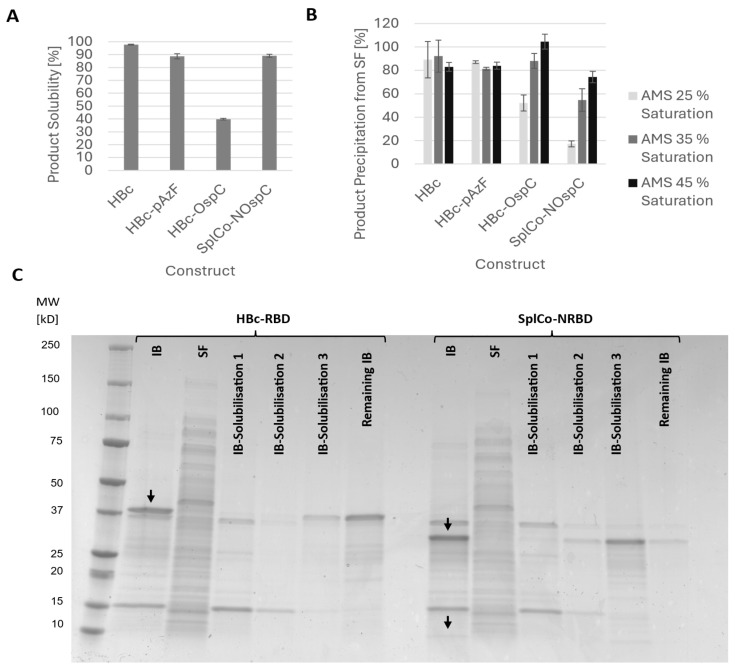
(**A**) Comparison of product recovery in the soluble fraction (SF) labeled as % solubility for the protein constructs HBc, HBc-*p*AzF, HBc-OspC and SplCo-NOspC after identical cultivation as previously described. Cell harvests of the same volume were enzymatically treated and mechanically disrupted and SF and IB fraction separately applied for SDS-PAGE and densitometrically evaluated for product content. (**B**) Comparison of precipitation efficiency for determination of apparent hydrophobicity of the same constructs from the resulting SF after cell disruption. Varying percentages of ammonia sulfate (AMS) saturation were used and resulting precipitates reconstituted and applied in SDS-PAGE for densitometric evaluation. Non-precipitated SF served as reference. Error bars represent standard deviation from mean value (biological triplicates). (**C**) SDS-PAA gel (Coomassie, greyscale) of solubilization samples for HBc-RBD and SplCo-NRBD. Black arrows indicate respective product band initially found in the IB fractions to serve as reference. Insoluble material after treatment with varying extraction buffer compositions was reconstituted in cell disruption buffer.

HBc was almost completely soluble in cell homogenate, and HBc-*p*AzF only differed slightly. The solubility of OspC antigen fusions depended on the HBc fusion partner and comprised only 40% of the contiguous fusion into the MIR of HBc; however, it comprised 90% of the CoreN fusion (Figure 3A). The RBD fusions were insoluble in cell homogenate, irrespective of the HBc fusion partner, and were recovered exclusively in the inclusion body (IB) fraction. Therefore, solubilization from the IB fraction was evaluated. The IBs were treated with various buffer compositions, but only the addition of 6 M urea under highly alkaline conditions resulted in the recovery of the soluble product. The majority of SplCo-NRBD could be solubilized under these conditions, whereas HBc-RBD remained mostly insoluble (Figure 3C). Previous experiments have shown additional incompatibilities with the proposed purification strategy, such as low binding in AEX and rapid product aggregation. Therefore, both RBD fusion variants were excluded from further purification experiments.

### 2.3. Evaluation of Platform-Based Purification Processes Applied to Genetic Fusions

#### 2.3.1. Native Purification of VLPs

**Figure 4:**
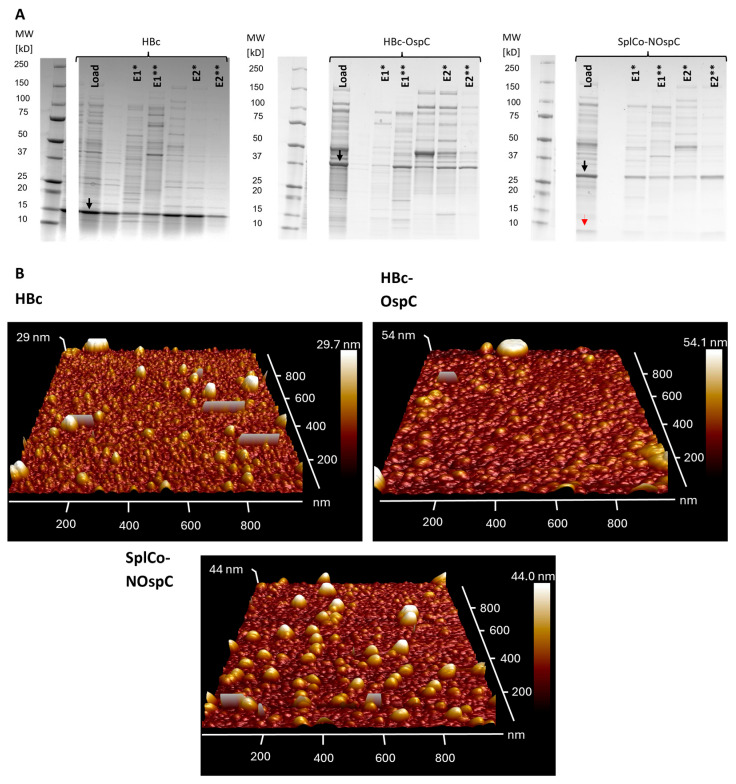
(**A**) SDS-PAA gels (Coomassie, greyscale) of non-dissociative AEX Load samples and collected fractions in varying dilutions dependent normalized by fraction volume. Black arrows highlight product bands of HBc, HBc-OspC and CoreN-OspC, while red arrow highlights CoreC product band as found in load sample to serve as reference. Separate fractions were characterized by two major UV signal peaks eluting at the same conductivity values during the gradient as E1 (15–18 mS/cm) and E2 (20–25 mS/cm) and applied in two parts as the first (*) and second (**) half of each peak. Marker lanes were taken from the same gels and added in identical image dimensions. (**B**) AFM height recordings in 3D visualization across a selected area of the mica surface from E2 fractions (pooled from both separately collected halves) with color scales indicating maximal recorded particle sizes for each construct. They were generated through the analysis software from amplitude data of the measurements. Particles appear as half-spheres in the 3D visualization due to the inaccessibility of the cantilever to the sample bottom. Rectangular shapes spread out across the tapping direction (along the *x*-axis) are caused by noise like electrical interferences during the measurement.

Ammonia sulfate (AMS) precipitation was the initial purification step, providing a crude separation of proteins from other host-related contaminants. To optimize product recovery while minimizing host protein carryover, apparent product hydrophobicity was assessed by adjusting different percentages of AMS. Lower hydrophobicity was observed for HBc-OspC and the CoreN-OspC fusions (Figure 3B). Thus, AMS precipitation was carried out uniformly using 45% saturation. The reconstituted precipitates were desalted via dialysis and used in AEX in a native state, presumably in VLP conformation. Similarly to neat HBc, both OspC fusion variants were detected in multiple elution fractions. Two main elution fractions, E1 and E2, were identified based on the associated conductivity ranges of 15 to 18 mS/cm and 20 to 25 mS/cm (Figure S1A). In all fractions except E2 of HBc, host cell proteins were abundant according to SDS-PAGE, indicating limited purification efficiency (Figure 4A). AFM analysis of the E2 fractions of HBc and the OspC fusions revealed spherical structures within the expected dimensions (Figure 4B). Varying maximum heights were suspected to be caused by overlaying particles, so size measurements were performed along the planar cross-section of each particle. Size measurements of a representative selection of particles (*n* = 30) resulted in mean diameters of 31.6 nm (±5.29 nm) for HBc and 39.3 nm (±5.14 nm) and 32.0 nm (±5.65 nm) for HBc-OspC and SplCo-NOspC, respectively.

**Figure 5:**
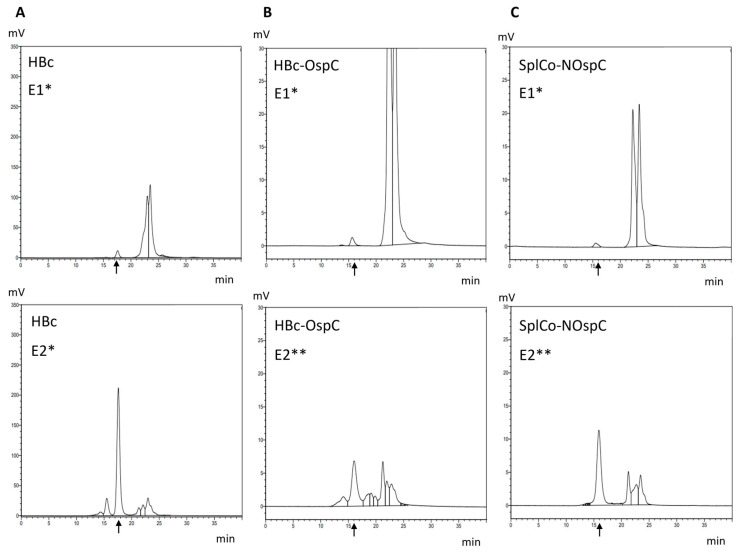
SEC Chromatograms of samples from native purification of either the first (*) or second half (**) of E1 and E2. Black arrows indicate the expected retention time of VLPs of the respective construct, which were validated during method development: (**A**) HBc, t_ret_ = 17.5 min; (**B**) HBc-OspC, t_ret_ = 15.8 min; (**C**) SplCo-NOspC, t_ret_ = 16.0 min.

To further confirm the presence of VLPs and to investigate a possible increase in the hydrodynamic radius due to the surface-presented OspC antigen, HPLC-SEC was performed as described. Retention times shifted from 17.5 min for HBc (Figure 5A) to 15.8 min and 16.0 min for HBc-OspC and SplCo-NOspC, the E2 fractions, while the E1 fractions showed few VLPs in HPLC-SEC.

#### 2.3.2. Dissociative Purification of Dimers and Reassembly into VLPs

**Figure 6:**
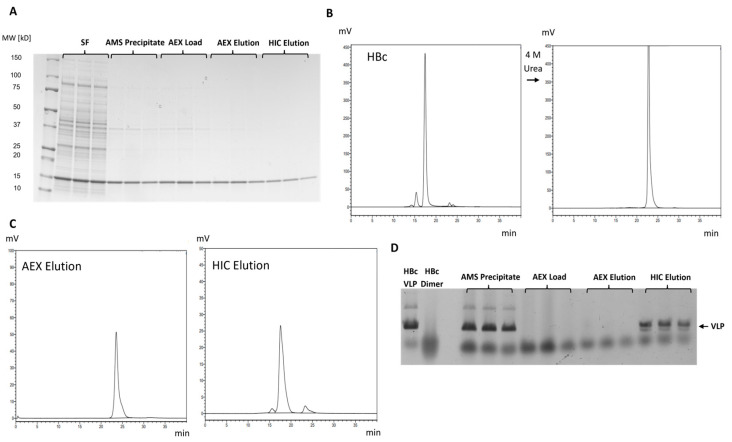
Process analytics from dissociative purification of HBc. (**A**) SDS-PAA gel (Coomassie, gray scale) of samples taken after subsequent process steps after identical cultivation and processing of triplicates. (**B**) SEC Chromatograms of purified HBc before and after incubation with 4 M Urea to validate dissociation of VLPs (t_ret_ = 17.5 min) into dimers (t_ret_ = 23 min). (**C**) SEC Chromatograms of AEX Elution and HIC Elution samples. (**D**) Native agarose gel (Coomassie, gray scale) showing change in migration behavior of purified HBc Control as VLP and Dimer (dissociated sample after incubation with 4 M Urea) and samples taken after subsequent process steps after identical cultivation and processing of triplicates.

The complete purification process, including disassembly and AEX and HIC-assisted reassembly, was first performed in triplicate with HBc, revealing a strong reduction in contaminants, as shown by SDS-PAGE (Figure 6A). Disassembly of an HBc VLP sample through incubation with 4 M urea was verified using HPLC-SEC, resulting in a retention time shift from 17.5 min to 23.3 min (Figure 6B). Applying this treatment prior to AEX, followed by elution in the presence of 2 M urea and an increasing salt concentration, resulted in a more homogeneous elution profile (Figure S1B). No unbound HBc was found in the flow-through fraction prior to elution. The only signal observed in the AEX elution sample was found at 23.3 min (Figure 6C), indicating no unintended reassembly of HBc under eluting salt concentrations when lowering the urea concentration from 4 M to 2 M during AEX. After HIC, the signal shifted back toward the VLP-related retention time. Disassembly and reassembly during the process were further validated by native agarose gel electrophoresis (NAGE) (Figure 6D). Dissociated dimers migrate diffusely through the highly porous gel, as observed for the AEX load and elution samples. Due to their increased size, VLPs have limited diffusivity and behave as more distinct protein bands with a decreased migration distance, further confirming successful reassembly in the HIC elution. The appearance of an additional band in HIC elution samples, slightly below the main VLP band, comparable to the control, was attributed to symmetry variations during reassembly (Higher-Band T = 4; Lower-Band T = 3 [35]). However, applying this process to HBc-OspC and SplCo-NOspC proved unsuccessful, as no binding was achieved in HIC. Additional reassembly attempts on the eluted fusions after AEX via AMS precipitation under increasing hydrophobic conditions showed no product recovery.

### 2.4. Transfer of Purification Strategies to HBc-pAzF

**Figure 7:**
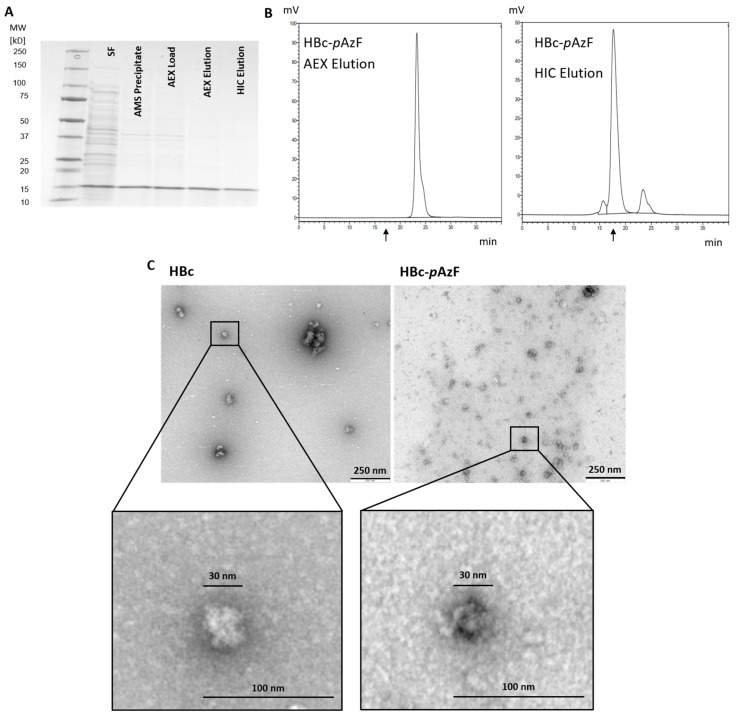
(**A**) SDS-PAA gel (Coomassie, gray scale) of samples taken during process purification of HBc-*p*AzF. (**B**) SEC Chromatograms of AEX Elution and HIC Elution of HBc-*p*AzF. Black arrows at expected retention time for HBc VLPs (tret = 17.5 min). (**C**) TEM Images of purified HBc and HBc-*p*AzF, with enlarged image sections of highlighted isolated particles, identifiable as a differently contrasted area compared to the image background with the expected particle size. The 30 nm sized markers were determined according to the respective scale of each image.

Evaluation of the apparent hydrophobicity of HBc-*p*AzF showed no difference compared with unmodified HBc, even when using 25% AMS saturation (Figure 3B). Therefore, the lower concentration was used for purification. This led to a visible decrease in co-precipitated host cell proteins, detectable in SDS-PAGE (Figure 7A), when comparing the load samples to those prepared at 45% saturation (Figure 4A). By loading HBc-*p*AzF onto AEX in its native state, supposedly in VLP conformation, the product could not be eluted from the column (Figure S1A). In dissociative purification, HBc-*p*AzF behaved similarly to HBc, and SDS-PAGE showed a significant increase in lane-purity over the course of purification (Figure 7A). As no detectable contaminating protein bands were present in the final HIC elution sample, a protein purity approaching 100% is assumed. Homogeneous binding and elution in AEX were observed (Figure S1B), and quantitative reassembly of the disassembled dimers after HIC was confirmed by SEC (Figure 7C). Final reassembly degrees, evaluated as the total peak area percentage of the VLP peak, reached 91.5% (±0.97%) for HBc and 84.2% (±5.1%) for HBc-*p*AzF. DLS measurements revealed highly uniform particle distributions for both VLP preparations, with polydispersity indices of 0.01. Z-averaged diameters of the main population fractions for HBc and HBc-*p*AzF were calculated as 38.0 nm and 49.4 nm, respectively. This difference in particle size could not be confirmed by SEC, as only a slight shift in retention time from 17.4 min to 17.7 min was detected. Purified samples containing HBc and HBc-*p*AzF showed spherical structures with expected diameters of approximately 30 nm in TEM (Figure 7C), identifiable as VLPs.

### 2.5. Application of HBc-pAzF in Click Reactions

#### 2.5.1. Qualitative Evaluation of CuAAC and SPAAC on Purified VLPs

**Figure 8:**
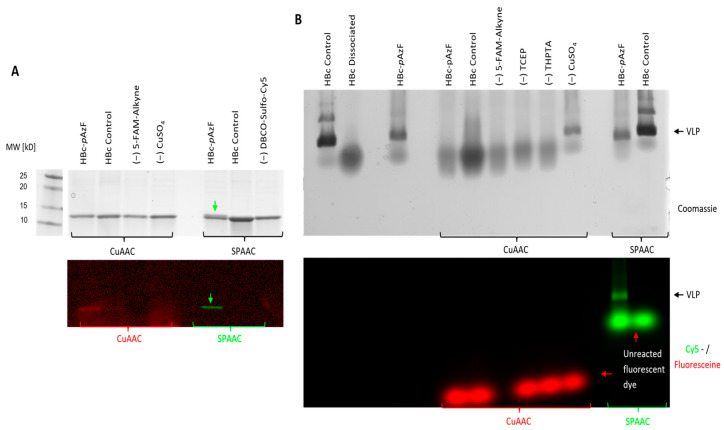
(**A**) SDS-PAA gel and (**B**) native agarose gel after click-reactions CuAAC and SPAAC with fluorescent dies. Lanes labeled “HBc-*p*AzF” contained the whole reaction mix, further lanes varied in either HBc replacing HBc-*p*AzF as negative control, or the absence (−) of certain reaction components. Green arrows indicate upwards-shifted product band after conjugation to DBCO-Sulfo-Cy5 in SPAAC. Red arrows highlight position of unreacted fluorescent dye. Marker lane was taken from the same gels and added in identical image dimensions. Protein visualization by Coomassie staining (gray scale) and multichannel detection of fluorescence before Coomassie staining. Red channel: fluorescein-detection; green channel: Cy5-detection.

Qualitative reactivity assessment of HBc-*p*AzF in CuAAC and SPAAC was performed by incubating a VLP-containing purified sample with the fluorescent dyes 5-FAM-Alkyne and DBCO-Sulfo-Cy5, respectively, along with additional reaction components for CuAAC, as described. CuAAC showed no change in band migration behavior in SDS-PAGE, whereas SPAAC led to a partial bandshift toward a higher molecular weight in the HBc-*p*AzF band (Figure 8A). Fluorescence activation of the same gel at wavelengths specific to both fluorescent dyes in a multichannel setting showed co-migration with the HBc-*p*AzF-band, indicating successful conjugation. Since all control lanes showed no fluorescence, the conjugation could be identified as specific, based on the respective mechanisms. When applied in NAGE, the particle stability of HBc-*p*AzF seemed to be disrupted by the reaction conditions of CuAAC (Figure 8B). Only the control sample, in which the catalyst precursor CuSO_4_ was excluded from the reaction mixture, showed a distinguishable particle band comparable to the HBc-control. All other combinations showed diffusive protein clouds, similar to the dissociation control, which was incubated with 4 M urea. Therefore, the copper salt was identified as the cause of VLP disruption. Co-migration of the fluorescent dye could not be observed, indicating low reaction efficiencies below the detectable limit. During SPAAC, the particle band was visible for both HBc variants, and only HBc-*p*AzF showed additional co-migration of the fluorescent dye, confirming the specificity of the conjugation with no disruption of VLP stability. Based on these findings, CuAAC was discarded as a suitable application for VLP functionalization, and further experiments focused on the efficiency of SPAAC.

#### 2.5.2. Analytical Evaluation of Particle Stability After SPAAC

**Figure 9:**
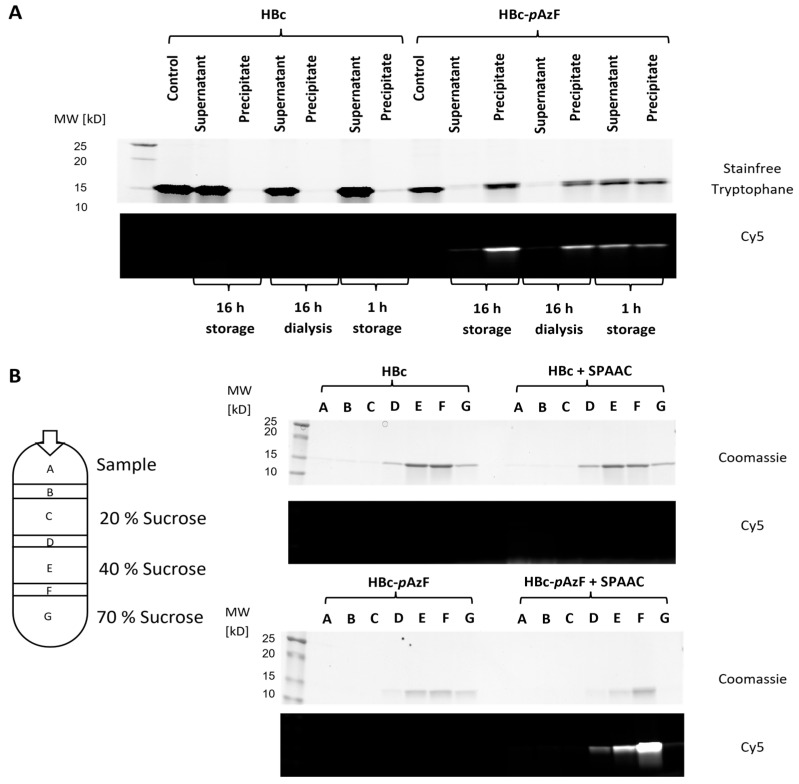
(**A**) SDS-PAA gel of purified HBc and HBc-*p*AzF after SPAAC and subsequent storage at 6 °C for one or 16 h or dialysis against TN-300 for 16 h at 6 °C (MWCO = 20 kDa). Lanes labeled “Control” were not subjected to SPAAC. Supernatants were separated after storage by centrifugation and precipitates were constituted in TN-300 in the same volume. Single-channel detection of Stainfree-fluorescence and Cy5-Fluorescence. (**B**) SDS-PAA gels of harvested fractions after SUC of HBc and HBc-*p*AzF. SPAAC of both constructs was conducted prior to centrifugation. Location of the sucrose layers with their respective concentrations and boundary layers are indicated by the diagram on the left and designated A, B, C, D, E, F and G. For each construct, these fractions were loaded on a SDS-Gel, as shown on the right. Protein visualization by Coomassie staining (gray scale) and single-channel detection of Cy5-Fluorescence before Coomassie staining.

After the SPAAC of the VLPs, visible colored precipitates formed at the bottom of the reaction tubes, and it was unclear whether particle agglomeration or protein aggregation had occurred. To verify the product content of these precipitates, samples were stored after SPAAC for 1 h at room temperature or 16 h at 6 °C, or they were dialyzed at 6 °C for 16 h to remove unreacted fluorescent dye. They were separated via centrifugation, and both precipitates and supernatants were analyzed using SDS-PAGE, alongside identically treated unmodified HBc (Figure 9A). The previously observed double band of HBc-*p*AzF indicated the presence of both reacted and unreacted monomers in the precipitated fractions. Fluorescence activation confirmed the co-precipitation of the reacted fluorescent dye, while unreacted DBCO-Sulfo-Cy5 appeared to remain in a soluble state. The difference in product distribution between 1 h storage and 16 h storage suggests slowed agglomeration kinetics rather than abrupt protein aggregation due to conformational disruptions by SPAAC.

VLP integrity after the SPAAC of HBc-*p*AzF was further investigated using SUC. SDS-PAGE analysis of the harvested fractions showed accumulation of the target protein in the 40 % (*w*/*v*) sucrose layer and the boundary layer to 70% (*w*/*v*) sucrose, and successful SPAAC was confirmed again through fluorescence activation of the gels (Figure 9B). The combination of HBc-*p*AzF and SPAAC showed a distribution shift toward the boundary layer, but no protein was detected in the 70% sucrose layer. Combined with the unchanged electrophoretic behavior of the VLP-DBCO-Sulfo-Cy5 conjugates, no protein aggregation could be derived, and agglomeration due to the hydrophobic nature of the fluorescent dyes appeared more likely.

#### 2.5.3. Quantitative Evaluation of SPAAC Reactivity During Purification

**Figure 10:**
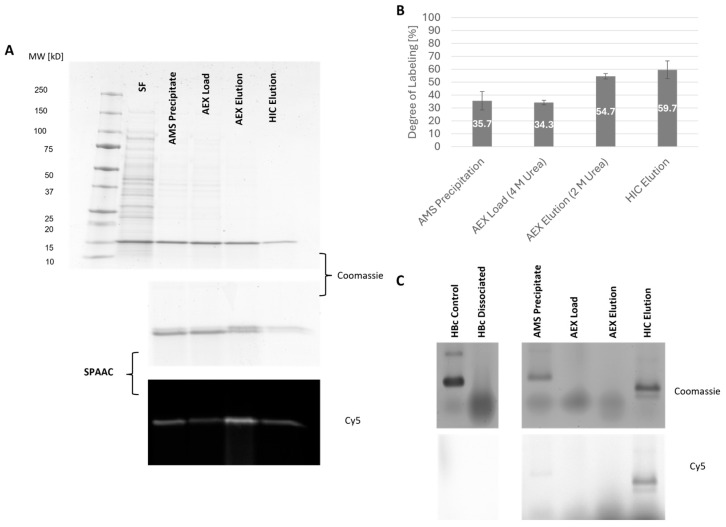
Evaluation of SPAAC reaction efficiency of samples taken during the dissociating purification process of HBc-*p*AzF. (**A**) SDS-PAA gel of SDS performed before and after SPAAC of the samples. (**B**) Degree of labeling evaluated by densitometric band comparison of upper protein band. Error bars represent standard deviation from mean value (biological triplicates). (**C**) Native agarose gel. Protein visualization by Coomassie staining (gray scale) and single-channel detection of Cy5-Fluorescence before Coomassie staining.

Chromatographic purification of HBc-*p*AzF resulted in the most noticeable increase in protein purity after AEX (Figure 10A). The previously reported bandshift, as well as the resulting fluorescence after SPAAC with DBCO-Sulfo-Cy5, indicated the increasing reactivity of HBc-*p*AzF samples along the purification process. Densitometric quantification of the shifted protein bands and comparison to the unreacted monomers showed an improved degree of labeling (Figure 10B). The dissociation of the particles after AMS precipitation with urea alone did not appear to have an effect. However, AEX also resulted in the strongest increase, along with improved protein purity. The efficiency of labeling remained high after reassembling the dimers into VLPs via HIC, suggesting little effect on the labeling equilibrium. Notably, only the assembled VLPs exhibited detectable fluorescence after NAGE, possibly due to the higher local focusing in a defined band compared with the diffusive clouds of the dissociated dimers (Figure 10C). The final labeling degree of approximately 59.7% (±6.94%) indicates full coverage of each protein dimer and, therefore, each surface-exposed protein-spike per VLP. The actual spatial distribution of the reacted fluorescent dye across the VLP surface was not investigated.

## 3. Discussion

### 3.1. Unfavorable Property Changes Caused by Genetic Fusions Limit Scalable Purification Strategies

While our results confirm the advantages of the SplitCore approach for OspC [22] and the identification of VLPs, they also highlight the remaining issues of genetic fusions in yielding chimeric VLPs containing heterologous antigens as vaccine candidates when applied to the SARS-CoV-2 spike protein RBD. The OspC fusions showed increased solubility using the SplitCore approach, but applying it to the SARS-CoV-2 RBD resulted in a poorly soluble product, requiring strong denaturing conditions for solubilization. AEX of the OspC fusions in their native state revealed remaining impurities and heterogeneous product elution behavior, comparable to unmodified HBc. Considering the comparable visible band intensities across the elution fractions, a separation via assembly state can be assumed. Adopting this purification approach would result in high product losses, as densitometric evaluation indicated a product distribution of approximately 50% in the presumed dimeric conformation found in E1. By contrast, dissociative conditions during purification of HBc resulted in a singular elution fraction during AEX, presumably indicating complete dimeric recovery of both HBc and the SplCo variants. Additionally, HBc showed highly efficient VLP-assembly during HIC. This could not be replicated for the OspC fusion variants, as no reassembly was achieved after dissociative AEX. Subsequent AMS precipitation using 45% AMS saturation was also tested as an alternative to the milder hydrophobic conditions of HIC, but to no success. In previous work, HBc-RBD was successfully purified via cation exchange chromatography under dissociative conditions. After removing urea through dialysis, product aggregation occurred when lowering the pH below 10.5. Aberrant structures observable in TEM (Figure S2) align with previous findings about the disrupted assembly competence of HBc after exposure to 6 M urea [14], which was necessary for solubilization of HBc-RBD and SplCo-NRBD. No further experiments on the complete disassembly–AEX–reassembly process for SplCo-NRBD were conducted. Therefore, correct protein folding and assembly competence appeared to be impaired by fusion variants tested in this study. An equilibrium between hydrophobic and electrostatic interactions has been identified as a key factor driving particle assembly [36]. Modifying the surface-exposed region of HBc, and thus its charge distribution along the insertion site, already introduces variations based on the fusion partner.

### 3.2. Singular Introduction of nnAA at MIR Retains the Advantages of Dissociative Purification

The minimal modification introduced by the exchange of Ala80 with nnAA *p*AzF led to only minor variations in solubility, hydrophobicity, and particle assembly, as expected. The targeted integration of *p*AzF using the pEVOL-plasmid showed no influence on product conformation as a VLP, with only a slight decrease in productivity. This could be attributed to the increased cellular energy demand required to maintain a secondary expression plasmid, combined with the additional required antibiotic as a selection marker. HBc-*p*AzF was also the only modified construct that facilitated the application of the disassembly–reassembly approach during purification. The purification process was replicated in a larger-scale setup, following the cultivation of the HBc expression strain in fed-batch fermentations. This resulted in protein purities of 99.5% in SDS-PAGE, VLP peak areas in SEC of up to 98.5%, and a final VLP yield of over 600 mg/L culture volume. As the results show only small differences in the behavior of HBc-*p*AzF compared with HBc, similar outcomes can be expected.

### 3.3. Effects of AAC Reaction Mechanisms on Particle Stability and Their Applicability to the HBc-Platform

Previous studies have shown successful functionalization of VLPs with nnAAs and copper-catalyzed click-chemistry [29]. However, the use of the methionine analogue AHA as the reactive group required modifications regarding the assembly domain of HBc and was a limitation that led to particle instability. While the application of CuAAC resulted in detectable conjugation with the fluorescent probe, the catalyst-bearing salt CuSO_4_ had a dissociative effect, independent of the cycloaddition itself. Interactions between the Cu(II) ion in solution and the particle surface may have led to particle disassembly, as dissociation also occurred in the absence of the reducing agent, TCEP. Denaturing effects of the high Cu^2+^ concentrations have also been observed in previous applications of CuAAC for protein labeling [33]. Studies addressing the antiviral activity of copper have found that Cu^2+^ has an increasing influence on the stability of charged proteins [37], which aligns with the aforementioned importance of surface charge on the HBc VLP for particle stability. Targeted mutations could successfully reduce or fully remove surface charge and introduce additional disulfide bonds to increase particle stability [38]. Applying these to the protein design of HBc-*p*AzF could help identify the underlying mechanism and enable the applicability of the highly reactive CuAAC reaction to HBc VLPs. SPAAC did not demonstrate a disruptive effect on VLP stability, and therefore, the formation of the 1,2,3-triazole ring can be excluded as a potential cause. The observable increase in sedimentation of the reacted VLPs was investigated and found to be attributable to slow agglomeration, rather than protein aggregation. This phenomenon could be driven by the high hydrophobicity of the fluorescent dye; however, it remains uncertain whether VLP–antigen conjugations caused by SPAAC would yield similar behavior.

### 3.4. Surface Exposure of the Reactive Azide Sidechain of HBc-pAzF Leads to High Accessibility

The improved and consistent reactivity of HBc-*p*AzF after AEX in SPAAC suggests the removal of contaminants that block the accessibility of the *p*AzF group through ionic interactions. It serves as an indirect indicator of the effectiveness of this additional purification step, although the residual protein purity was already high after the precipitation step. Notably, AEX under non-dissociative conditions yielded no elution of HBc-*p*AzF, presumably due to the ionic interactions of the high local density of the azide ion on the VLP surface, leading to very strong binding to the chromatography medium. In combination with a comparably high SPAAC efficiency before and after the VLP reassembly during HIC, this serves as an additional verification of the surface exposure of the reactive azide group from *p*AzF in this HBc variant, irrespective of its state of assembly.

It remains to be seen whether the presented findings are transferable for generating in vitro HBc–antigen conjugations. The high diffusivity of the small fluorescent probes used in this study may have enabled easier accessibility to the azide group on the VLP. Larger antigens, such as the SARS-CoV-2 spike RBD, may be subject to repulsive effects from the particle surface, leading to further challenges for efficient conjugation. Successful protein–protein conjugations using SPAAC have been reported [39] but showed slow conjugation rates at temperatures that may degrade certain antigens.

## 4. Materials and Methods

### 4.1. Genes and Cloning

The plasmids pRSF-T7_HBc149opt-TetPrRep and pRSF-T7_splHBc149opt-TetPrRep, along with the coding sequence of the OspC antigen, were provided by the working group of Professor Dr. Michael Nassal. They carry open reading frames (ORFs) to encode the truncated Hepatitis B core antigen (HBc) and the corresponding SplitCore (SplCo) proteins CoreN and CoreC. The plasmid backbone served as a vector for the protein expression plasmids constructed in this study. All expression plasmids feature an inducible T7 promoter upstream of the ORF, the pRSF origin of replication, and a kanamycin resistance gene for selection. The ORFs for the protein variants HBc-OspC, HBc-RBD, and HBc-*p*AzF were optimized for expression in *E. coli* and synthesized using GeneArt (Thermofisher, Waltham, MA, USA). These sequences were cloned into the expression vector via PCR amplification of the insert and vector, with suitable overlaps added to facilitate seamless integration via assembly cloning. To clone the SplCo-plasmids, the HBc-OspC and HBc-RBD fusion inserts were amplified up to the C-terminal end of the respective foreign antigen. The vector included an additional stop-codon, a ribosome binding site, and the CoreC sequence to enable the bicistronic expression of the CoreN fusions and CoreC. Primers were synthesized using Microsynth Seqlab (Göttingen, Germany). The NEBuilder^®^ HiFi DNA Assembly Cloning Kit (New England Biolabs, Ipswich, MA, USA) was then used to assemble and transform the fragments, which were then transformed into *E. coli* NEB 10-beta cells for cloning (New England Biolabs, MA, USA). The bacteria were cultured at 37 °C in lysogeny broth (LB) [40], supplemented with 50 mg/L kanamycin (Applichem, Darmstadt, Germany). The production plasmids were subsequently cloned into *E. coli* BL21(DE3) (Novagen, Madison, WI, USA) following chemical competency treatment according to internal protocols. Shortly after growth in LB, cells were subjected to centrifugation and resuspended in ice-cold 0.1 M CaCl_2_. After incubation on ice for up to one hour, the process was repeated using 85 mM CaCl_2_ and 15% glycerol. These stocks were then stored at −80 °C. For transformation, competent bacterial stocks were thawed on ice, and appropriate amounts of plasmid were added; they were then subjected to heat-shock treatment at 42 °C for a defined time. After recovery in a complex medium, the cells were plated onto LB agar containing 50 mg/L kanamycin for selection. The plasmid pEVOL-*p*AzF was a gift from Peter Schultz (Addgene plasmid # 31186; http://n2t.net/addgene:31186 (accessed on 30 June 2025); RRID: Addgene_31186 [41], Watertown, MA, USA) and subsequently transformed into the strain expressing HBc-*p*AzF. Selection plates and cultures for the resulting strain were additionally supplemented with 50 mg/L chloramphenicol, as pEVOL-*p*AzF confers chloramphenicol resistance.

### 4.2. Protein Constructs

A truncated variant of HBc (HBV genotype D, serotype ayw; accession no.: CAA 24706) ending at Val149 served as the scaffold for all protein modifications. The MIR of the surface-exposed loop spans Leu76 to Arg82. The SplCo system consists of two separate polypeptides, CoreN and CoreC, which represent the respective N-terminal and C-terminal portions of HBc, split between Pro79 and Ala80. In the fusion proteins HBc-OspC, HBc-RBD, Core-NOspC, and Core-NRBD (the latter two combined with unmodified CoreC in SplCo-OspC or SplCo-RBD), the inserted antigen sequence is flanked by N- and C-terminal polyglycine-linkers. In HBc-OspC and HBc-RBD, Pro79 was deleted to minimize steric hindrance at the insertion site. For HBc-*p*AzF, the nnAA was incorporated by introducing the amber stop-codon in place of Ala80. Stop-codons at the C-terminal end of the protein did not involve the amber stop-codon, in accordance with the pEVOL-*p*AzF system [31].

### 4.3. Protein Expression

Glycerol stocks of the BL21(DE3) strains were streaked onto LB agar plates containing 50 mg/L kanamycin (and 50 mg/L chloramphenicol for the HBc-*p*AzF-expressing strain) and incubated overnight at 37 °C. Precultures in liquid LB medium were then grown at 37 °C with shaking for up to 18 h, typically reaching an optical density of approximately 5.0 at 600 nm per mL (OD600/mL) after 7 h. Expression cultures were then inoculated to an OD600/mL of 0.05 or 0.2 (for HBc-RBD and SplCo-NRBD) in the respective media. Two culture media were evaluated for the expression of HBc-*p*AzF. PYMD-0.2 is a complex medium adapted from ZYM-5052 [42], designed to yield high cell densities in autoinduction cultures. To enable controlled induction, lactose was substituted with glucose. M9 is a defined minimal medium containing 10 mM MgSO_4_, 1 mM CaCl_2_, 2% glucose, 0.6% Na_2_HPO_4_, 0.3% KH_2_PO_4_, 1% NH_4_CL, and 0.05% NaCl. Cultures were grown at 37 °C to an OD600/mL of 0.6 to 1.2 per mL. Protein expression was induced by adding an induction mix containing 1 mM IPTG, 0.2% Arabinose, and 1 mM *p*AzF (only for HBc-*p*AzF), followed by incubation at 30 °C for 4 h (HBc-RBD and SplCo-NRBD) or 17 h (HBc, HBc-OspC, SplCo-NOspC, HBc-*p*AzF). For native purification experiments, the autoinduction Medium ZYM-5052 [43] was utilized, with soy peptone replacing N-Z-amines, and without the need for preincubation at 37 °C or a separate induction step.

Cells were harvested via centrifugation, washed in TN-300 Buffer (25 mM Tris-HCl, 300 mM NaCl, pH 7.5), and stored at –20 °C until further use. To evaluate expression efficiency, chemical lysis of cells was performed via reconstitution in SDS-PAGE sample buffer.

### 4.4. Cell Disruption

Cell disruption was performed by setting the disruption volume (DV) to a biomass concentration of approximately 30 OD600/mL for solubility and precipitation efficiency quantification, or 130 OD600/mL for purification. Harvested cells were reconstituted in TN-300 buffer, followed by the addition of 100 mg/L Lysozyme, 1 mM PMSF, and 0.05 % (*v*/*v*) Triton X-100. The suspension was incubated on ice for 30 min. Subsequently, nuclease digestion was initiated by adding 20 U/mL Benzonase^®^ (Merck, Darmstadt, Germany) and 1 mM MgCl_2_ during incubation at 30 °C for 30 min. Mechanical disruption was then performed by shaking the suspension with an equal volume of glass beads (0.1–0.2 mm) at 2500 rpm in 1.5 mL reaction tubes for 30 min at 6 °C. The glass beads were then washed with 2 DV TN-300, and the resulting cell homogenate was clarified via centrifugation. The supernatant (soluble fraction, SF) and precipitated cell debris (inclusion body fraction, IB) were collected and stored separately. For solubility quantification, the IB fraction was reconstituted in 3 DV TN-300 for SDS-PAGE analysis.

In the solubilization experiments for HBc-RBD and SplCo-NRBD, the IB fraction was reconstituted in three separate extraction buffers, with centrifugation and separation from the preceding step’s supernatant. Solubilization buffer 1: 50 mM Tris-HCl, 150 mM NaCl, 1 mM EDTA, 4 M urea, 1% (*v*/*v*) Triton X-100, 2 mM DTT, and a pH of 8.5. Solubilization buffer 2: 20 mM Na_2_CO_3_, 150 mM NaCl, 1 mM EDTA, 4 M urea, 2 mM DTT, and a pH of 10.5. Solubilization buffer 3 was identical to solubilization buffer 2, but the urea and DTT concentrations were raised to 6 M and 5 mM, respectively.

### 4.5. Protein Precipitation

A saturated solution of 4.1 M ammonium sulfate (AMS) was defined as 100% AMS saturation and added to SF, followed by an incubation at 6 °C for 30 min. AMS saturation was varied between 25% and 45% to quantify precipitation efficiency. For native purification of HBc, HBc-OspC, and SplCo-NOspC, 35% AMS saturation was used, while 25% AMS saturation was employed for the dissociative purification of HBc and HBc-*p*AzF. Precipitated protein was recovered via centrifugation, and the resulting supernatants were discarded. The precipitate was reconstituted in half of the initial SF volume in resolvation buffer (20 mM Tris-HCl, pH 8.5). It was incubated at room temperature for 30 min, followed by a second centrifugation. Supernatants were collected and dialyzed against resolvation buffer using Slide-a-lyzer™ cassettes (Thermofisher) with a molecular weight cut-off of 20 kDa. Dialysis was performed at 6 °C for 18 h.

### 4.6. Anion Exchange Chromatography

The resolvation buffer used after AMS precipitation served as the mobile phase in AEX. For elution, it was supplemented with 1.0 M NaCl. For dissociative purification, wash and elution buffers were supplemented with 2 M urea to prevent reassociation of HBc dimers. The stationary phase employed for native purification was Toyopearl™ DEAE 650 m (Tosoh Bioscience, Griesheim, Germany), packed into a self-packed column and a ready-to-use HiTrap™ DEAE FF column (Cytiva, Marlborough, MA, USA) for dissociative purification. Chromatography was performed on an Äkta Pure™ purification system (Cytiva, MA, USA) at room temperature. The linear flow rates were 115 cm/h for the Toyopearl™ column and 350 cm/h for the Cytiva™ column. Before the sample application, columns were equilibrated using 5 column volumes (CVs) of load buffer. After the sample load, an additional 5 CVs of load buffer were applied as a wash step. Elution was performed using a linear gradient from 0% to 50% (*v*/*v*) elution buffer. For native purification, 15 CVs were employed, whereas for dissociative purification, 5 CVs were used. Eluted fractions were collected and separated based on UV absorption peaks at 280 nm.

### 4.7. Hydrophobic Interaction Chromatography

HIC was carried out using a CIMmultus^®^ OH 1 mL monolithic column (Sartorius, Göttingen, Germany) with a channel size of 6 µm, installed on the Äkta pure™ System, as described for AEX. The mobile phases consisted of TN-300 buffer (Mobile Phase A) and 2 M potassium phosphate (Mobile Phase B) at pH 7.5. Urea-containing load samples were five-fold diluted in Mobile Phase A and loaded for HIC via 1:1 inline dilution in Mobile Phase B, resulting in load conditions of 1 M potassium phosphate in TN-300. After the sample application, the column was washed in 50% (*v*/*v*) Mobile Phase B in Mobile Phase A. Step elution was performed in Mobile Phase A. Eluted fractions were collected based on UV absorption, as previously described.

### 4.8. Protein Analysis

Protein monomers and product-containing fractions were identified via sodium dodecyl sulfate polyacrylamide gel electrophoresis (SDS-PAGE) using the Criterion™ Cell System (Bio-Rad Laboratories, Hercules, CA, USA) with TGX Stain-Free™ precast gels (4–20%) and the corresponding sample buffer, as previously described [44]. A reducing agent of 17% (*v*/*v*) β-mercaptoethanol was used in sample preparation. Gels were run at 250 V for 25 min. Strain-free fluorescence—caused by tryptophane reacting with trihalogen compounds in the stain-free system [45]—was detected using a Chemidoc™ MP detection chamber (Bio-Rad Laboratories, CA, USA), and gels were subsequently stained with Coomassie dye [43]. Densitometric evaluation of band intensity was performed using the ImageLab™ software package, version 6.1.0 (Bio-Rad Laboratories). Protein concentrations were determined using the Bradford Assay [46], with BSA as the protein standard.

### 4.9. Particle Analysis

Particle detection was conducted via analytical size exclusion chromatography (SEC) on a modular high-performance liquid chromatography (HPLC) system (Shimadzu LC-20 series, Kyoto, Japan) with UV–Vis detection set to a wavelength of 280 nm. A BioBasic SEC 1000 column (Thermofisher; 15 mL column volume, 1000 Å pore size) was used at a flow rate of 0.5 mL/min, and TN-300 buffer was used as a mobile phase. Native agarose gel electrophoresis (NAGE) was carried out in gels containing 1% (*w*/*v*) agarose in TRIS–borate–EDTA buffer. Electrophoresis was performed at 100 V for 90 min. Gels were either directly activated for the detection of fluorescent dyes or stained with GelCode™ Blue Stain (Thermofisher, MA, USA). Sucrose gradient ultracentrifugation (SUC) was performed using three different sucrose solutions (70% (*w*/*v*), 40% (*w*/*v*), and 20% (*w*/*v*) in TN-300), layered from top to bottom. The sample was applied to the top, and centrifugation was performed using an Optima™ L90K centrifuge (rotor type: 70.1 Ti, tubes: 16 mm × 76 mm, Beckman Coulter, Brea, CA, USA) at 51,000 rpm for 90 min. Fractions of each layer, including phase boundaries, were collected from top to bottom and analyzed via SDS-PAGE. Particle size and homogeneity were evaluated using dynamic light scattering (DLS) and transmission electron microscopy (TEM), as described in [47]. Particle morphology was investigated via atomic force microscopy (AFM) imaging of the nanoparticles. A MICA disk V1 grade was initially cleaved thrice. After the application of 20 µL of the nanoparticle suspension (undiluted, approximately 0.2 mg/mL protein), the suspension was allowed to dry completely on the MICA surface. Subsequently, 20 µL of Aqua Millipore (Burlington, MA, USA) was added to the previously dried spot and allowed to rest for around 30 min, preventing drying in a covered Petri dish. The sample was then washed thrice with around 100 µL of Aqua Millipore to remove suspension remnants. Finally, the washed sample was dried under constant air flow (hand bellows). For AFM imaging of nanoparticle samples in air, intermittent contact mode imaging was used employing a Bruker Dimension 3100 AFM (Bruker, Karlsruhe, Germany) equipped with HQ:NSC14/Al BS cantilevers (MikroMasch, Sofia, Bulgaria). A free RMS amplitude of 0.6 to 0.8 V and an amplitude setpoint of around 0.5 V were used for stable imaging at a scanning rate of 0.5 Hz. Image data were corrected (flattening 0th and 1st order) and analyzed with Nanoscope Analysis 3.0 (Bruker, Karlsruhe, Germany).

### 4.10. Click-Chemistry Reactions

For CuAAC reactions, samples were mixed with 50 µM 5-FAM-Alkyn (Abs/Em = 490/513 nm, Jena Bioscience, Jena, Germany), 1 mM TCEP (Carl Roth, Karlsruhe, Germany), 0.17 mM THPTA (Baseclick, Munich, Germany), and 1 mM CuSO_4_. For SPAAC reactions, the samples were mixed with 50 µM DBCO-Sulfo-Cy5 (Abs/Em = 646/661 nm, Jena Bioscience, Jena, Germany). In both cases, reaction conditions were adjusted using a 10-fold TN-300-stock solution if necessary, and reaction mixtures were incubated at 25 °C for 60 min in the dark. After incubation, reaction mixtures were directly treated for analysis via SDS-PAGE or NAGE. Fluorophore detection followed in the Chemidoc™ MP detection chamber under preset detection parameters according to the manufacturer’s recommendations.

## 5. Conclusions

Our study confirmed the successful particle formation previously achieved with genetic antigen fusions using HBc VLPs as a surface presentation platform. These were designed via antigen insertion into the MIR of HBc and by utilizing SplitCore technology. However, this could not be transferred to the SARS-CoV-2 spike RBD, which proved incompatible as a fusion partner for both HBc and CoreN in the SplitCore approach due to insolubility. These efforts highlighted challenges in purification, such as decreased solubility and hydrophobicity, which are critical factors for efficient particle assembly in high yields. These issues are inherent to genetic protein fusions and require adaptations throughout the purification process. As an alternative approach, HBc was successfully labeled with the non-natural amino acid *p*AzF, enabling in vitro bio-orthogonal conjugation reactions, commonly referred to as click-chemistry. This modified HBc-*p*AzF variant was efficiently disassembled, purified as dimeric subunits, and reassembled to unmodified HBc with comparable efficiency and homogeneity. This approach facilitates simple and reproducible functionalization of HBc VLPs, contributing to the growing research interest in HBc as a modifiable VLP platform. Conjugation to fluorescent dyes serving as reaction partners for the copper-free bioconjugation reaction SPAAC showed promising results, with high reaction efficiencies and no obstruction of particle stability, in contrast to CuAAC. Generating HBc conjugates using this approach can be used in various applications, including antigen presentation in medical contexts, such as vaccines [48], or diagnostic approaches where the VLPs could serve as signal amplifiers [49].

## Data Availability

The datasets analyzed during the current study are available from the corresponding author on reasonable request.

## Supplementary Materials

The following supporting information can be downloaded at https://www.mdpi.com/article/10.3390/ijms262010036/s1.

## Abbreviations

The following abbreviations are used in this manuscript:

VLP	virus-like particle
OspC	outer surface protein C
RBD	receptor binding domain
HBc	hepatitis B core antigen
*p*AzF	*p*-azidophenylalanine
HBV	hepatitis B virus
AEX	anion exchange chromatography
SEC	size exclusion chromatography
SUC	sucrose cushion ultracentrifugation
HIC	hydrophobic interaction chromatography
MIR	major immunodominant region
CuAAC	copper-catalyzed azido-alkyne cycloaddition
nnAA	non-natural amino acids
SPAAC	strain-promoted azido-alkyne cycloaddition
SDS-PAGE	sodium dodecasulfate polyacrylamide gel electrophoresis
SDS-PAA	sodium dodecasulfate polyacrylamide
SplCo	SplitCore
ORF	open-reading frame
LB	lysogeny broth
OD600	optical density at a wavelength of 600 nm
TN-300	buffer containing 25 mM Tris, 300 mM sodium chloride at a pH of 7.5
DV	disruption volume
SF	soluble fraction
IB	inclusion body fraction
AMS	ammonium sulfate
CV	column volumes
HPLC	high pressure liquid chromatography
NAGE	native agarose gel electrophoresis
DLS	dynamic light scattering
TEM	transmission electron microscopy
AFM	atomic force microscopy
