# Supplementary material for: Modular Virus-like Particles for Antigen Presentation: Comparing Genetic Fusion and Click-Chemistry for Purification
Source: Int J Mol Sci. 2025 Oct 15;26(20):10036. doi: 10.3390/ijms262010036 (PMC12562343; doi:10.3390/ijms262010036)
Supplement: Supplementary file 1 [file ijms-26-10036-s001.zip › ijms-3805083-supplementary.pdf]

International Journal of
Molecular Sciences

Supplementary Material

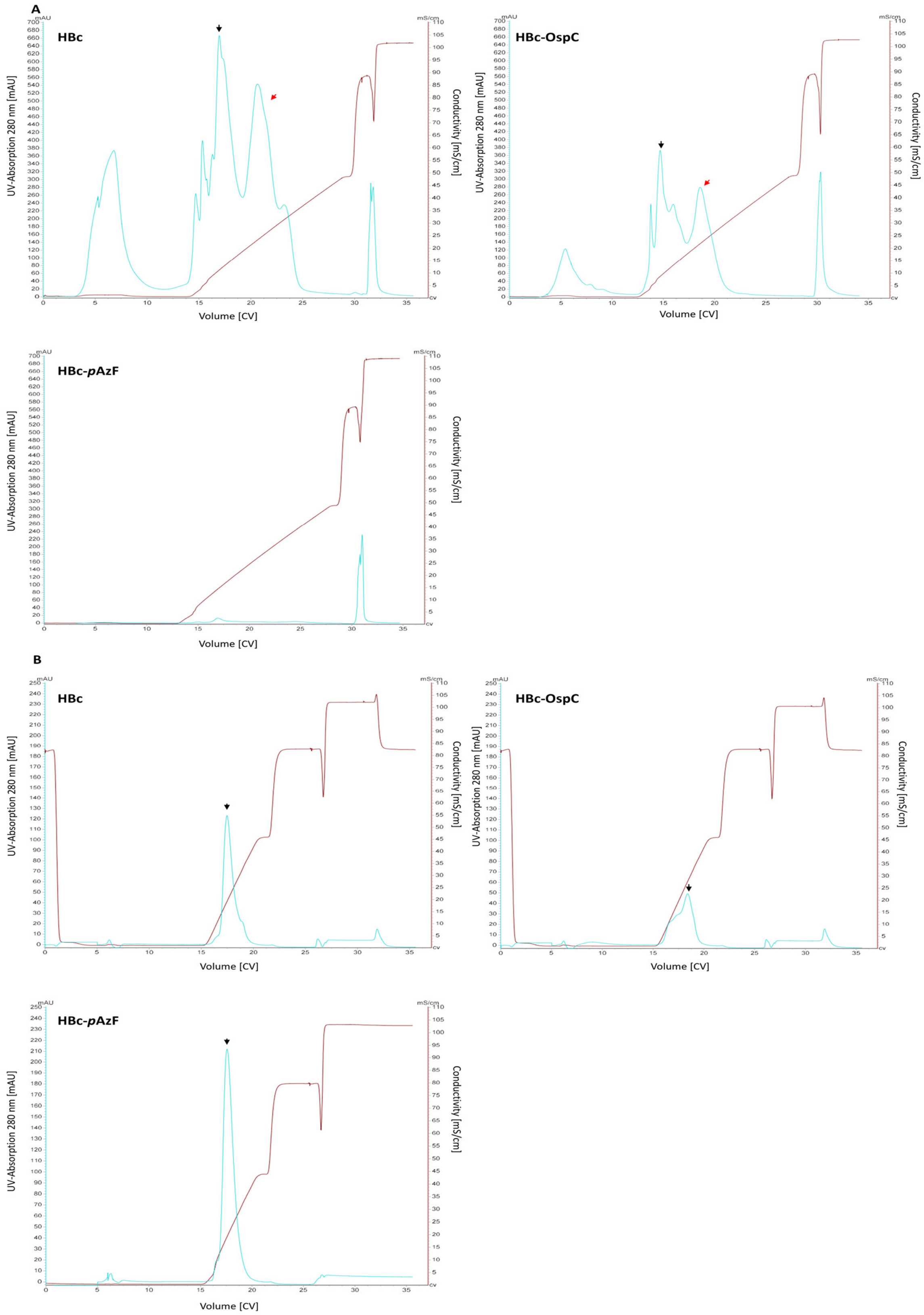

Figure S1. AEX Chromatograms of (A) non-dissociative and (B) dissociative purification of HBc, HBc-OspC and HBc-pAzF. Black arrows indicate first UV-Signal peaks with product content and

red arrows indicate second UV-Signal peaks with product content. For HBc-pAzF, there was no product found in non-dissociative elution. Variations in UV and conductivity signals before 5 CV and after 25 CV were due to manual variations for column cleaning.

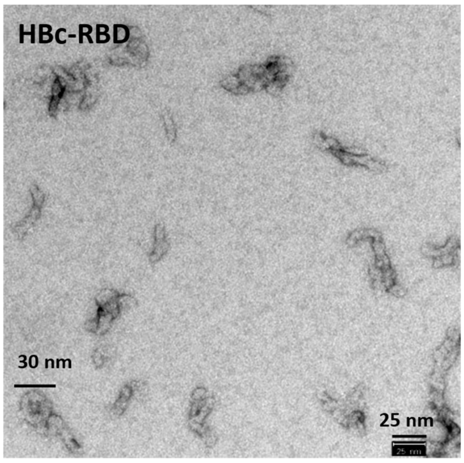

Figure S2. TEM Image of purified HBc-RBD. 30 nm - size marker on selected spherical structure visible in the recordings was measured according to the respective scale of each image.
